# Hypermethylation of the GATA binding protein 4 (GATA4) promoter in Chinese pediatric acute myeloid leukemia

**DOI:** 10.1186/s12885-015-1760-5

**Published:** 2015-10-21

**Authors:** Yan-Fang Tao, Fang Fang, Shao-Yan Hu, Jun Lu, Lan Cao, Wen-Li Zhao, Pei-Fang Xiao, Zhi-Heng Li, Na-Na Wang, Li-Xiao Xu, Xiao-Juan Du, Li-Chao Sun, Yan-Hong Li, Yi-Ping Li, Yun-Yun Xu, Jian Ni, Jian Wang, Xing Feng, Jian Pan

**Affiliations:** 1 Department of Hematology and Oncology, Childrens Hospital of Soochow University, Suzhou, China; 2 Department of Gastroenterology, the 5th Hospital of Chinese PLA, Yin chuan, China; 3 Department of Cell and Molecular Biology, Cancer Institute (Hospital), Chinese Academy of Medical Sciences, Peking Union Medical College, Beijing, China; 4 Translational Research Center, Second Hospital, The Second Clinical School, Nanjing Medical University, Nanjing, China

**Keywords:** GATA binding protein 4, Pediatric acute myeloid leukemia, Methylation, Tumor suppressor

## Abstract

**Background:**

Acute myeloid leukemia (AML) is the second-most common form of leukemia in children. Aberrant DNA methylation patterns are a characteristic feature of AML. GATA4 has been suggested to be a tumor suppressor gene regulated by promoter hypermethylation in various types of human cancers although the expression and promoter methylation of GATA4 in pediatric AML is still unclear.

**Methods:**

Transcriptional expression levels of GATA4 were evaluated by semi-quantitative and real-time PCR. Methylation status was investigated by methylation-specific PCR (MSP) and bisulfate genomic sequencing (BGS). The prognostic significance of GATA4 expression and promoter methylation was assessed in 105 cases of Chinese pediatric acute myeloid leukemia patients with clinical follow-up records.

**Results:**

MSP and BGS analysis showed that the GATA4 gene promoter is hypermethylated in AML cells, such as the HL-60 and MV4-11 human myeloid leukemia cell lines. 5-Aza treatment significantly upregulated GATA4 expression in HL-60 and MV4-11 cells. Aberrant methylation of GATA4 was observed in 15.0 % (3/20) of the normal bone marrow control samples compared to 56.2 % (59/105) of the pediatric AML samples. GATA4 transcript levels were significantly decreased in AML patients (33.06 ± 70.94; *P* = 0.011) compared to normal bone marrow/idiopathic thrombocytopenic purpura controls (116.76 ± 105.39). GATA4 promoter methylation was correlated with patient leukocyte counts (WBC, white blood cells) (*P* = 0.035) and minimal residual disease MRD (*P* = 0.031). Kaplan-Meier survival analysis revealed significantly shorter overall survival time in patients with GATA4 promoter methylation (*P* = 0.014).

**Conclusions:**

Epigenetic inactivation of GATA4 by promoter hypermethylation was observed in both AML cell lines and pediatric AML samples; our study implicates GATA4 as a putative tumor suppressor gene in pediatric AML. In addition, our findings imply that GATA4 promoter methylation is correlated with WBC and MRD. Kaplan-Meier survival analysis revealed significantly shorter overall survival in pediatric AML with GATA4 promoter methylation but multivariate analysis shows that it is not an independent factor. However, further research focusing on the mechanism of GATA4 in pediatric leukemia is required.

**Electronic supplementary material:**

The online version of this article (doi:10.1186/s12885-015-1760-5) contains supplementary material, which is available to authorized users.

## Background

Acute myeloid leukemia (AML) is a heterogeneous clonal disorder of hematopoietic progenitor cells, which lose the ability to differentiate normally and to respond to normal regulators of proliferation [1]. Pediatric AML comprises up to 20 % of all childhood leukemias. Epigenetic disturbances have been implicated in the development and pathogenesis of leukemia [2]. These include aberrations in methylation, which is a key epigenetic event responsible for enhanced proliferation and self-renewal, differentiation arrest, and impaired apoptosis of leukemic cells [3]. Several studies have evaluated genome-wide methylation in acute myeloid leukemia [4]. In AML, the presence of common methylation patterns in a few genes such as p15 and E-cadherin has been described independently by several groups across large patient cohorts [5, 6]. Progression from myelodysplastic syndrome to AML has also been associated with increased aberrant DNA methylation [7]. Identifying these aberrantly methylated genes may provide a better understanding of AML, thereby paving the way for the development of novel tumor markers and therapeutic targets.

In vertebrates, the existence of a covalent modification of the base cytosine in the context of CpG dinucleotides by addition of a methyl group to C-5 has been appreciated since the mid-70s [8]. The promoter regions of approximately 50 % of human genes contain regions of DNA with a cytosine and guanine content greater than expected (so-called CpG islands) that, once hypermethylated, mediate transcriptional silencing. The human genome consists of approximately 28 million CpGs, of which 60–80 % are normally 5-C methylated [9]. Approximately 10 % of CpGs occur in the context of CpG islands: CpG-rich regions which are on average 1 kilobase in size [9]. The following distinct roles in genomic methylation have been reported for DNMT isoforms: DNMT1 preferentially replicates already existing methylation patterns; DNMT3A and 3B are responsible for establishing de novo methylation. Abnormal expression of these methylation-related enzymes may disturb DNA methylation in pediatric AML. In cancer, aberrantly occurring DNA hypermethylation of these CpG islands, especially in tumor suppressor genes, is a well-established phenomenon, which occurs alongside a global loss of methylation, which in turn is associated with genomic instability [10, 11]. A common approach to the study of DNA methylation is to treat cells with 5-aza-2'-deoxycytidine (5-Aza) demethylation reagent. This epigenetic modifier inhibits DNA methyltransferase activity, resulting in DNA demethylation (hypomethylation); as such, treatment with 5-Aza can identify the genes that are inactivated by methylation.

Transcription factors of the GATA family are essential regulators of the specification and differentiation of numerous tissues. GATA factors typically bind to the element A/T GATA A/G to coordinate cellular maturation with proliferation arrest and cell survival. GATA4 is a member of the GATA family of zinc finger transcription factor, which regulates gene transcription by binding to GATA elements [12]. GATA4 was originally discovered as a regulator of cardiac development and subsequently identified as a major regulator of adult cardiac hypertrophy. GATA4 works in combination with other essential cardiac transcription factors as well, such as Nkx2-5 and Tbx5 [13]. GATA4 is expressed in both embryo and adult cardiomyocytes where it functions as a transcriptional regulator for many cardiac genes, and also regulates hypertrophic growth of the heart [14]. Mutations or defects in the GATA4 gene can lead to a variety of cardiac problems including congenital heart disease, abnormal ventral folding, and defects in the cardiac septum separating the atria and ventricles, and hypoplasia of the ventricular myocardium [15]. In addition to the heart, GATA4 plays important roles in the reproductive system, gastrointestinal system, respiratory system and cancer [16].

Numerous studies gave implicated GATA4 as a tumor suppressor gene involved in tumorigenesis in various types of human cancers. A previous investigation of the methylation status of GATA4 promoters by methylation-specific PCR in 99 glioblastoma patients showed that GATA4 was aberrantly methylated in 23.2 % of glioblastoma tumors, but not in normal brain [17]. In endometrioid carcinoma, GATA4 promoter methylation was detected in 81.5 % (44/54) of the carcinoma group and in none of the control group [18]. In ovarian cancer, methylation-specific PCR revealed GATA4 promoter methylation in 31.3 % (21/67) of specimens with ovarian cancer, and in none of the control ovarian tissue samples [19]. Furthermore, methylation of GATA4 is significantly higher in the ovarian cancer group compared with the control group [20]. Methylation of GATA4 was found in human gastric mucosa samples, including normal gastric biopsies, gastric dysplasia (low-grade gastric intraepithelial neoplasia) and paired sporadic gastric carcinomas (SGC) as well as the adjacent non-neoplastic gastric tissues.GATA4 methylation was frequently observed in SGCs (53.8 %) by MSP. Moreover, a high frequency of GATA-4 methylation was found in both gastric low-grade GIN (57.1 %) and indefinite for dysplasia (42.9 %). However,GATA4 methylation was detected only in 4/32 (12.5 %) of normal gastric biopsies. Epigenetic inactivation of GATA4 by methylation of CpG islands is an early frequent event during gastric carcinogenesis and is significantly correlated with *H. pylori* infection [21]. Promoter methylation of GATA4 was analyzed in colorectal tissue and fecal DNA from colorectal cancer patients and healthy controls using methylation-specific PCR. GATA4 methylation was observed in 70 % (63/90) of colorectal carcinomas and was independent of clinicopathologic features [22]. In glioblastoma multiforme (GBM), loss of GATA4 was observed in 58 % (94/163) of GBM operative samples and was found to be a negative survival prognostic marker [23]. Furthermore,GATA4 promoter methylation was detected in 67 % (42/63) of primary lung cancers [24]. In diffuse large B-cell lymphoma (DLBCL) GATA4 showed significant methylation in over 85 % of tumors [25].

Currently, the expression of GATA4 and the methylation status of its promoter in pediatric acute myeloid leukemia have not been reported. In this study, we have provided the first evidence of GATA4 methylation in two AML cell lines and pediatric myeloid leukemia samples. These data suggest that GATA4 may function as a tumor suppressor in pediatric acute myeloid leukemia.

## Methods

### Cell lines

Leukemia cell lines HL-60, MV4-11, U937, DAMI and K562 were obtained from the American Type Culture Collection (ATCC). CCRF, Raji, Jurkat, 697 and SHI-1 cell lines (gifts from Professor Wang Jian-Rong, The Cyrus Tang Hematology center of Soochow University). The entire cell lines were maintained at 37 °C in the RPMI 1640 (GibcoR, Life Technologies, Carlsbad, CA) supplemented with 10 % fetal bovine serum (Invitrogen, Life Technologies, Carlsbad, CA).

### Patients and samples

Bone marrow specimens were obtained at the time of diagnosis during routine clinical assessment of 105 pediatric patients with AML, who presented at the Department of Hematology and Oncology, Children's Hospital of Soochow University between 2006 and 2011. Research involving human subjects, human material, or human data, have been performed in accordance with the Declaration of Helsinki. Ethical approval was provided by the Children's Hospital of Soochow University Ethics Committee (No.SUEC2006-011 and No.SUEC2000-021), and informed consent was obtained from the parents or guardians. AML diagnosis was made in accordance with the revised French–American–British (FAB) classification. Additionally, bone marrow samples from 12 healthy donors and 8 patients with Idiopathic thrombocytopenic purpura (ITP) were analyzed as controls. Bone marrow mononuclear cells (BMNCs) were isolated using Ficoll solution within 2 h after bone marrow samples harvested and immediately subjected for the extraction of total RNA and genomic DNA.

### CD34 + cell purification

For CD34^+^cell selection, the Miltenyi immunoaffinity device (VarioMACS 130-046-703) was used according to the manufacturer’s instructions (Miltenyi Biotech, Auburn, CA). Briefly, the CD34^+^ cells are magnetically labeled with CD34 MicroBeads. Then, the cell suspension is loaded onto a MACSR Column which is placed in the magnetic field of a MACS Separator. The magnetically labeled CD34^+^ cells are retained within the column. The unlabeled cells run through; this cell fraction is thus depleted of CD34^+^ cells. After removing the column from the magnetic field, the magnetically retained CD34^+^ cells can be eluted as the positively selected cell fraction.

### Analysis of promoter methylation in pediatric AML by NimbleGen Human DNA Methylation arrays

Analysis of the methylation status of genes in five pediatric AML samples (M1, M2, M3, M4 and M5) and three NBM samples (N1, N2, and N3) using NimbleGen Human DNA Methylation arrays. NimbleGen Human DNA Methylation arrays Protocol: Step 1, Genomic DNA Extraction and Fragmentation, Genomic DNA (gDNA) was extracted from 8 samples using a DNeasy Blood & Tissue Kit (Qiagen, Fremont, CA). The purified gDNA was then quantified and quality assessed by nanodrop ND-1000. Step 2, Immunoprecipitation, 1 μg of sonicated genomic DNA was used for immunoprecipitation using a mouse monoclonal anti-5-methylcytosine antibody (Diagenode). For this, DNA was heat-denatured at 94 °C for 10 min, rapidly cooled on ice, and immunoprecipitated with 1 μL primary antibody overnight at 4 °C with rocking agitation in 400 μL immunoprecipitation buffer (0.5 % BSA in PBS). To recover the immunoprecipitated DNA fragments, 200 μL of anti-mouse IgG magnetic beads were added and incubated for an additional 2 h at 4 °C with agitation. After immunoprecipitation, a total of five immunoprecipitation washes were performed with ice-cold immunoprecipitation buffer. Washed beads were resuspended in TE buffer with 0.25 % SDS and 0.25 mg/mL proteinase K for 2 h at 65 °C and then allowed to cool down to room temperature. MeDIP DNA were purified using Qiagen MinElute columns (Qiagen). Step 3, Whole Genome Amplification (WGA). Step 4, DNA Labelling and Array Hybridization, the purified DNA was quantified using a nanodrop ND-1000. For DNA labelling, the NimbleGen Dual-Color DNA Labeling Kit was used according to the manufacturer’s guideline detailed in the NimbleGen MeDIP-chip protocol (Nimblegen Systems, Inc., Madison, WI, USA). Microarrays were hybridized at 42 °C during 16 to 20 h with Cy3/5 labelled DNA in Nimblegen hybridization buffer/ hybridization component A in a hybridization chamber (Hybridization System - Nimblegen Systems, Inc., Madison, WI, USA). For array hybridization, Roche NimbleGen's Promoter plus CpG Island array was used, which is a 385 k format array design containing 28,226 CpG Islands and all well-characterized Promoter regions (from about -800 bp to +200 bp of the TSSs) totally covered by ~385,000 probes. This NimbleGen Human DNA Methylation array analysis was performed by KangChen Bio-tech, Shanghai P.R. China.

### Sodium bisulphite modification of genomic DNA

High-molecular-weight genomic DNA was extracted from cell lines and biopsies by a conventional phenol/chloroform method. The sodium bisulphite modification procedure was as described with slight modification [26–28]. In brief, 600 ng of genomic DNA was denatured in 3 M NaOH for 15 min at 37 °C, then mixed with 2 volumes of 2 % low-melting-point agarose. Agarose/DNA mixtures were then pipetted into chilled mineral oil to form agarose beads. Aliquots of 200 μl of 5 M bisulphite solution (2.5 M sodium metabisulphite, 100 mM hydroquinone, both Sigma, USA) were added into each tube containing a single bead. The bisulphite reaction was then carried out by incubating the reaction mixture for 4 h at 50 °C in the dark. Treatments were stopped by equilibration against 1 ml of TE buffer, followed by desulphonation in 500 μl of 0.2 M NaOH. Finally, the beads were washed with 1 ml of TE buffer and directly used for PCR.

### Methylation-specific PCR

The methylation status of the GATA4 (NCBI Reference Sequence of GATA4 : NG_008177.2) promoter region was determined by methylation-specific PCR. Primers were designed with Methprimer design tool (http://www.urogene.org/methprimer/). Primers distinguishing unmethylated (U) and methylated (M) alleles were designed to amplify the sequence: GATA4 B M-forward: 5- TTTTTTAATTTTTGTTTGTATATCGT-3; GATA4 B M-reverse: 5- ACTACCTAACACTACCACCCTACGT-3; GATA4 B U-forward: 5- TTTTTTAATTTTTGTTTGTATATTGT-3; GATA4 B U-reverse: 5- CTACCTAACACTACCACCCTACATC-3.

Each PCR reaction contained 20 ng of sodium bisulphite-modified DNA, 250 pmol of each primer, 250 pmol deoxynucleoside triphosphate, 1 × PCR buffer, and one unit of ExTaq HS polymerase (Takara, Tokyo) in a final reaction volume of 20 μl. Cycling conditions were initial denaturation at 95 °C for 3 min, 40 cycles of 94 °C for 30 s, 65 °C (M) or 63 °C (U) for 30 s, and 72 °C for 30 s. For each set of methylation-specific PCR reactions, in vitro-methylated genomic DNA treated with sodium bisulphite served as a positive methylation control. PCR products were separated on 4 % agarose gels, stained with ethidium bromide and visualized under UV illumination. For cases with borderline results, PCR analyses were repeated.

### Bisulfite genomic sequencing

Bisulfite genomic sequencing (BGS) was performed as previously described. BGS primers were from +682 to +904 including 17 CpGs. GATA4 F: 5- GGATTGAATGTTTTTTTGGAAGTT-3 and GATA4 R: 5- CCTCCTTTCCTCAACCTAATAACA-3. Amplified BGS products were TA-cloned; and five to six randomly chosen colonies were sequenced. DNA sequences were analyzed with QUMA Analyzer. (http://quma.cdb.riken.jp/).

### Leukemia cell cells treated with 5-aza-2'-deoxycytidine

De-methylation was induced with 5-aza-dC (5-Aza, Sigma-Aldrich, St Louis, MO, USA) treatment at a concentration that induced de-methylation of the DNA without killing the cells. Culture media for HL-60 and MV4-11 cells contained 5 μM 5-Aza. DNA and RNA were extracted after 72 h of 5-Aza treatment for the following analysis.

### Quantitative reverse-transcription PCR for GATA4

Quantitative real-time PCR was performed to determine the expression levels of GATA4 genes. Total RNA was reverse transcribed using the Reverse Transcription Kit, according to the manufacturer's protocol (Applied Biosystems Inc., Foster City, CA). The real time PCR primers used to quantify GAPDH expression were: F: 5′-AGAAGGCTGGGGCTCATTTG-3′ and R: 5′-AGGGGCCATCCACAGTCTTC-3′ and for GATA4 were: F: 5′- TAGCCCCACAGTTGACACAC-3′ and R: 5′- GTCCTGCACAGCCTGCC −3′. Real-time PCR analysis was according to the MIQE Guidelines and performed in a total volume of 20 μl including 1 μl of cDNA, primers (0.2 mM each) and 10 μl of SYBR Green mix (Roche). Reactions were run on an Lightcycler 480 (Roche) using universal thermal cycling parameters (95 °C for 5 min, 45 cycles of 10 s at 95 °C, 20 s at 60 °C and 15 s at 72 °C; followed by a melting curve: 10 s at 95 °C, 60 s at 60 °C and continued melting). The results were obtained using the sequence detection software of the Lightcycler 480 and analyzed using Microsoft Excel. For quality control purposes, melting curves were acquired for all samples. The comparative Ct method was used to quantify gene expression. The target gene expression level was normalized to expression of the housekeeping gene glyceraldehyde 3-phosphate dehydrogenase (GAPDH) within the same sample (−⊿Ct), the relative expression of GATA4 was calculated with 10^6^ *Log2(-⊿Ct ).

### Western blot analysis

Western blot analysis was introduced before [29]. Cellular proteins were extracted in 40 mM Tris–HCl (pH 7.4) containing 150 mM NaCl and 1 % (v/v) Triton X-100, supplemented with protease inhibitors. Equal amounts of protein were resolved on 12 % SDS-PAGE gels, and then transferred to a PVDF membrane (Millipore, Bedford, MA). Blots were blocked and then probed with Polyclonal Goat IgG antibodies against GATA4 (1:1000, R&D. Minneapolis, MN) and GAPDH (1:5000, Sigma, St. Louis, MO). After three times’ washing, blots were then incubated with horseradish peroxidase (HRP) conjugated secondary antibodies and visualized by enhanced chemiluminescence kit (Pierce, Rockford, IL). Protein bands were visualized after exposure of the membrane to Kodak X-ray film.

### Statistical analysis

SPSS v11.5 (SPSS Inc., Chicago, IL) was used for statistical analysis. Data are presented as means ± standard deviation. Group t-test was used to compare the expression of GATA4 between DMSO group and 5-Aza group. Statistical significance between methylated sample data and clinical pathological features of AML patients were analyzed by Pearson chi-square test or Fisher's exact test. Statistical significance of GATA4 expression among NBM and pediatric AML groups was determined using one-way ANOVA. A p <0.05 was considered statistically significant.

## Results and discussion

### The GATA4 promoter is hypermethylated in AML cells

**Fig. 1:**
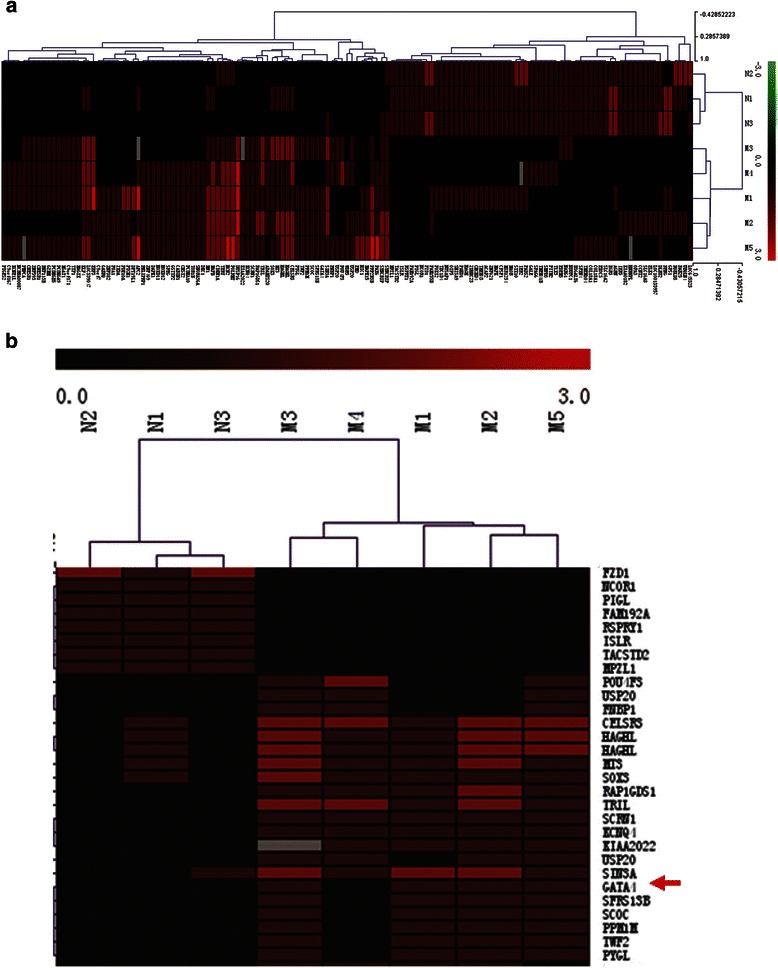
Promoter methylation analysis of pediatric AML with NimbleGen Human DNA Methylation Arrays. **a** Analysis of the methylation status of genes in four pediatric AML samples (M1, M2, M3, M4 and M5) and three NBM samples (N1, N2, and N3) using NimbleGen Human DNA Methylation Arrays. Each *red box* represents the number of methylation peaks (PeakScore) overlapping the promoter region for the corresponding miRNA. The PeakScore is defined as the average -log10 (*P*-value) from probes within the peak. The scores reflect the probability of positive methylation enrichment. **b** DNA methylation array analysis showing significant methylation of the GATA4 promoter in AML samples (4/5), and unmethylated in NBM samples (0/3)

The correlation between aberrant methylation and downregulation of GATA4 has been extensively documented in numerous cancers and cell lines; these are discussed in the Background. However, the methylation status of GATA4 in the blood system, particular in pediatric AML, has not been reported to date. Our analyses of promoter methylation in pediatric AML, using NimbleGen Human DNA Methylation 385 K Promoter plus CpG Island arrays, indicated that the GATA4 promoter is hypermethylated in AML (Fig. 1a). The GATA4 promoter was hypermethylated in 80 % (4/5) of pediatric AML samples and 0 % (0/3) of normal bone marrow samples (Additional file 1) .

**Fig. 2:**
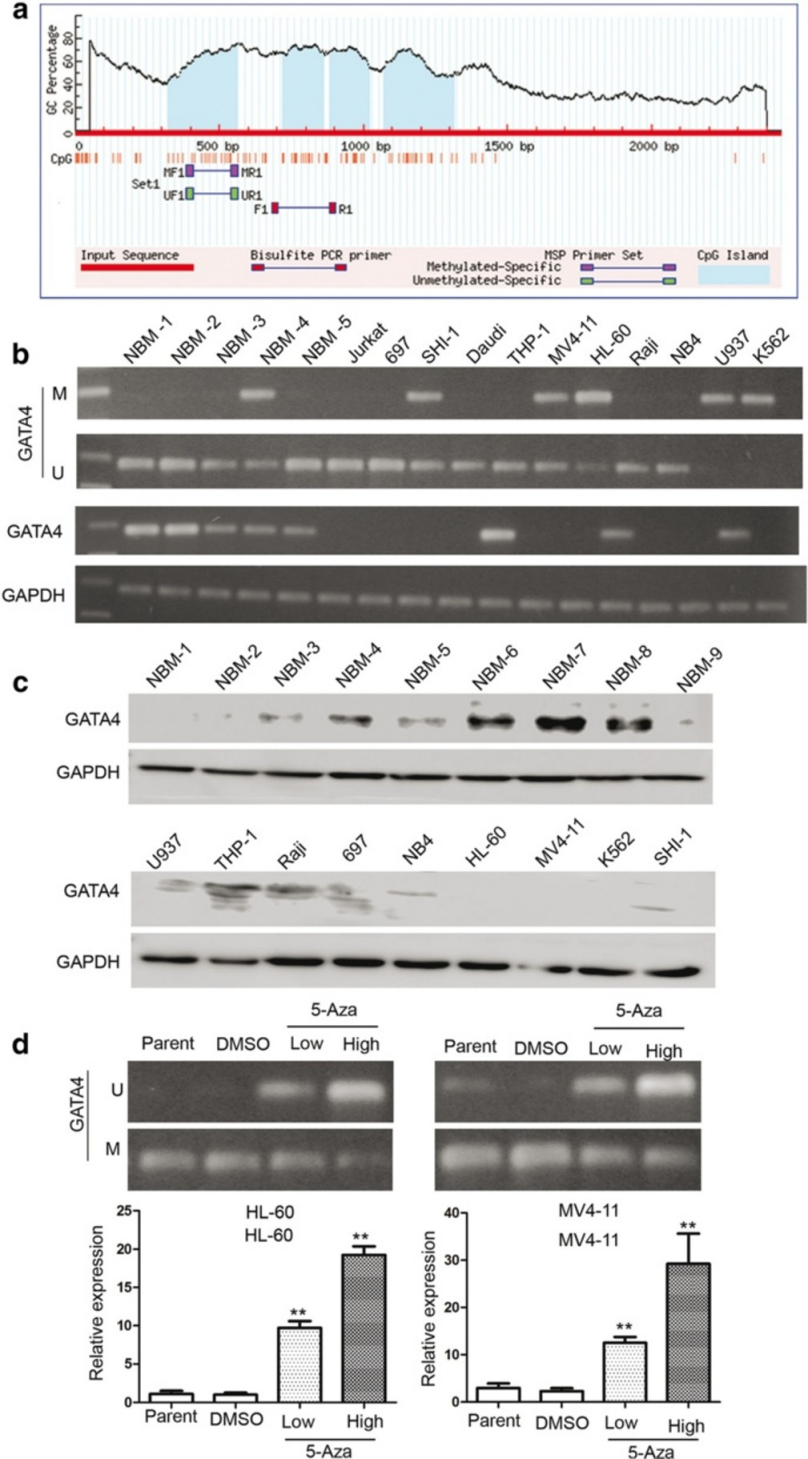
The GATA4 promoter is methylated in AML cell lines. **a** Four CpG island regions can be identified in the promoter of GATA4. **b** MSP analysis of the methylation status of GATA4 in leukemia cell lines showing hypermethylation in 5/11 cell lines. M and U represent MSP results using primer sets for methylated and unmethylated GATA4 genes, respectively. **c** Western blot analysis the expression of GATA4 in 9 NBM samples and 9 leukemia cell lines. **d** The GATA4 transcript level is upregulated in cells treated with 5-Aza compared to DMSO: 19.2-fold in HL-60 cells (5-Aza: 19.23 vs. DMSO: 1.00; *P* = 0.003); 12.5-fold in MV4-11 cells (5-Aza: 29.23 vs. DMSO: 2.33; *P* = 0.05)

Subsequent analyses of the GATA4 promoter sequence identified four CpG islands (Fig. 2a). Methylation-specific PCR (MSP) assays were performed to detect the methylation status of the GATA4 promoter in 11 leukemia cell lines. The MSP primers were designed using MethPrimer (http://www.urogene.org/cgi-bin/methprimer/methprimer.cgi ) to encompass the CpG islands of the GATA4 promoter identified in Fig. 2a. Our results showed that the GATA4 promoter was hypermethylated in five leukemia cell lines, especially in SHI-1, HL-60, MV4-11,U937 and K562 cells); and unmethylated in the other cell lines (Fig. 2b). The results of RT-PCR analysis of the expression of GATA4 is presented in Fig. 2b; GATA4 expression was detected in only three cell lines (THP-1, Raji and U937), indicating that downregulation of GATA4 in AML cells is a common phenomenon. Figure 2c showed that expression of GATA4 in leukemia cell lines is significantly lower than NBM. 6/9 NBM samples with obvious expression of GATA4 and in leukemia cell lines GATA4 only can be detected in THP-1 and Raji cells. To confirm methylation of the GATA4 promoter, we treated the leukemia cell lines with the demethylation reagent 5-Aza. Our results showed that 5-Aza treatment significantly upregulated GATA4 expression. As shown in Fig. 2d, GATA4 expression was upregulated 19.2-fold in HL-60 cells (5-Aza: 19.23 vs. DMSO: 1.00; *P* = 0.003) and 12.5-fold in MV4-11 cells (5-Aza: 29.23 vs. DMSO: 2.33; *P* = 0.05). These results were supported by the MSP analyses, which also showed a change in the methylation status of the GATA4 promoter after 5-Aza treatment. In summary, these results showed that the GATA4 promoter was consistently and significantly methylated in the HL-60, MV4-11, SHI-1, U937 and K562 human myeloid leukemia cell lines. Based on these findings, we hypothesized that the promoter of GATA4 is methylated in pediatric AML patients.

### The GATA4 promoter is methylated in pediatric AML patients

**Fig. 3:**
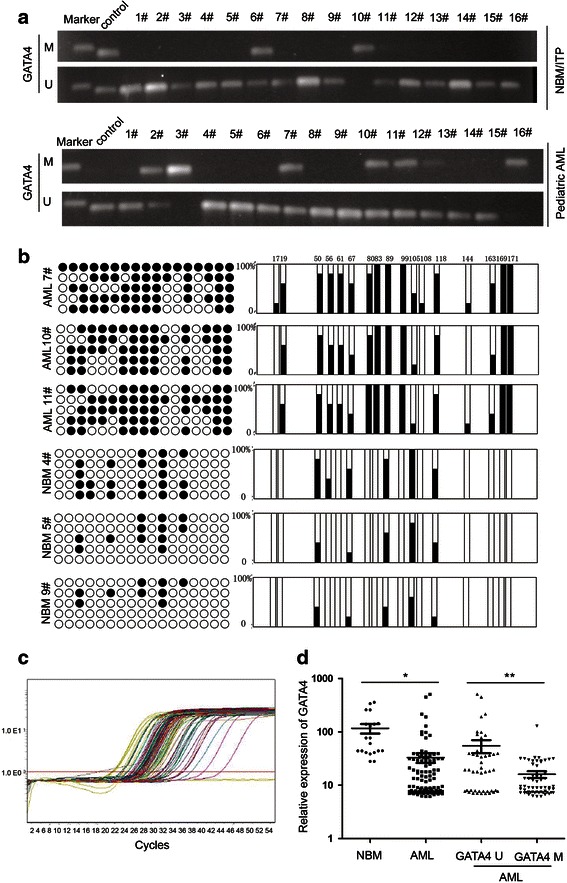
GATA4 is inactivated by promoter hypermethylation in pediatric AML. **a** MSP analysis of the methylation status of GATA4 shows aberrant methylation in pediatric AML samples compared to NBM/ITP control samples. Aberrant methylation of GATA4 was observed in 15.0 % (3/20) of the NBM control samples compared to 56.2 % (59/105) of the pediatric AML samples. **b** Three NBM samples and three AML samples were analyzed by BSG. The results showed that the CpG islands in the GATA4 promoter were methylated in the AML samples (69.4, 58.8, and 62.4 % in AML7#, AML10#, and AML11#, respectively). In contrast, the CpG islands of the GATA4 promoter in the NBM samples were unmethylated (24.7, 14.1, and 10.6 % in NBM4#, NBM5#, and NBM9#, respectively). **c** The transcript levels of GATA4 were examined in 105 pediatric AML patients by real-time PCR. **d** GATA4 expression was significantly decreased in 105 AML patients (33.06 ± 70.94; *P* =0.011) compared to 20 NBM/ITP controls (116.76 ± 105.39); AML patients with GATA4 promoter methylation (16.02 ± 17.59, n = 59) showed lower GATA4 transcript levels compared to those without GATA4 promoter methylation (54.92 ± 101.80, *P* <0.001; *n* = 46)

We next examined the GATA4 promoter methylation status in pediatric AML samples and NBM/ITP (normal bone marrow/idiopathicthrombocytopenic purpura) control samples. Aberrant GATA4 promoter methylation was observed in 15.0 % (3/20) of the NBM control samples compared to 56.2 % (59/105) of the pediatric AML samples (Fig. 3a). Three NBM samples and three AML samples were further analyzed by BSG (Fig. 3b). The results showed that the CpG islands in the GATA4 promoter were methylated in the AML samples (69.4, 58.8, and 62.4 % in AML7#, AML10#, and AML11#, respectively). In contrast, the CpG islands of the GATA4 promoter in the NBM samples were unmethylated (24.7, 14.1, and 10.6 % in NBM4#, NBM5#, and NBM9#, respectively). These results were supported by MSP assays.

### Expression of GATA4 is downregulated with promoter methylation in Chinese pediatric acute myeloid leukemia

The transcript levels of GATA4 were examined in 105 pediatric AML patients by real-time PCR (Fig. 3c). As shown in Fig. 3d, GATA4 expression was significantly decreased in 105 AML patients (33.06 ± 70.94; *P* = 0.011) compared to that in 20 NBM/ITP controls (116.76 ± 105.39). Figure 3d shows that patients with GATA4 promoter methylation (16.02 ± 17.59, n = 59) exhibited lower GATA4 transcript levels compared to those without GATA4 promoter methylation (54.92 ± 101.80, *P* < 0.01; n = 46). Furthermore, AML patients with and without GATA4 promoter methylation showed significantly lower GATA4 transcript levels compared to those in controls.

**Fig. 4:**
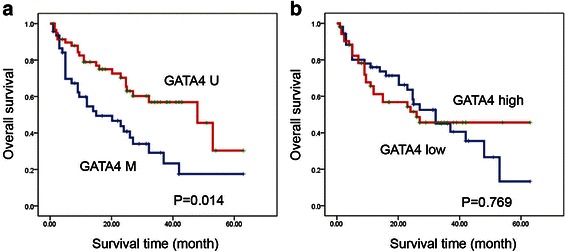
GATA4 promoter methylation correlates with poor survival in Chinese pediatric acute myeloid leukemia. **a** Kaplan-Meier survival analysis in pediatric AML samples with GATA4 promoter methylation status (*P* = 0.014). **b** Kaplan-Meier survival analysis in pediatric AML samples with GATA4 expression (*P* = 0.769)

The prognostic significance of GATA4 expression was assessed in 105 cases of Chinese pediatric acute myeloid leukemia patients with clinical follow-up records. There was no significant association with GATA4 expression and patient age, sex, FAB (French–American–British classification) or cytogenetics (Table 1). Kaplan-Meier survival analysis of 105 pediatric acute myeloid leukemia patients revealed almost identical survival times for patients with GATA4 high or low expressing tumors (*P* = 0.769, Table 3 and Fig. 4b). Furthermore, multivariate analysis revealed that GATA4 expression was not an independent prognostic factor in pediatric AML (*P* = 0.096, Table 4).

**Table 1:** Association of GATA4 expression with clinico-pathological characteristics in 105 pediatric AML samples

Clinical variables	No. of patients	GATA4 expression (n)		*P*
		Low	High	
Sex				
Male	42	24	18	0.265
Female	63	29	34	
Age (years)				
< 6	60	32	28	0.499
≥ 6	45	21	24	
Leukocyte (/μl)				
> 10,000	61	32	29	0.632
≤ 10,000	44	21	23	
FAB				
M1–M6	93	49	44	0.207
M7	12	4	8	
Cytogenetics				
Favorable	50	20	30	0.077
Intermediate	27	18	9	
Unfavorable	28	15	13	
MRD				
< 0.25 %	49	23	26	0.498
≥ 0.25 %	56	30	26

### GATA4 promoter methylation correlates with poor survival in Chinese pediatric acute myeloid leukemia

The prognostic significance of GATA4 promoter methylation was also assessed in 105 cases of Chinese pediatric acute myeloid leukemia patients with clinical follow-up records. Table 2 shows GATA4 promoter methylation was correlated with leukocyte counts (*P* = 0.035) and MRD (*P* = 0.031). Table 2 also shows there were no significant differences in clinical features, such as sex, age, FAB or cytogenetics between patients with and without GATA4 promoter methylation. Kaplan-Meier survival analysis revealed significantly shorter overall survival times in patients with GATA4 promoter methylation (*P* = 0.014, Table 3 and Fig. 4a). Furthermore, multivariate analysis revealed that GATA4 promoter methylation was not an independent prognostic factor in pediatric AML (*P* = 0.170, Table 4).

**Table 2:** Association of GATA4 promoter methylation with clinico-pathological characteristics in 105 pediatric AML samples

Clinical variables	No. of patients	GATA4 methylation (n)		P
		Negative	Positive	
Sex				
Male	42	21	21	0.297
Female	63	25	38	
Age (years)				
< 6	60	28	32	0.496
≥ 6	45	18	27	
Leukocyte (/μl)				
> 10,000	61	32	29	0.035
≤ 10,000	44	14	30	
FAB				
M1–M6	93	38	55	0.090
M7	12	8	4	
Cytogenetics				
Favorable	50	16	34	0.062
Intermediate	27	14	13	
Unfavorable	28	16	12	
MRD				
< 0.25 %	49	16	33	0.031
≥ 0.25 %	56	30	26

**Table 3:** Association of GATA4 expression/promoter methylation with Kaplan-Meier survival in 105 pediatric AML samples

Variable	No. of patients	Over survival	*P*
		Median ± SE	
Cytogenetics			
Favorable	50	46.664 ± 3.717	<0.001
Intermediate	27	29.220 ± 3.188	
Unfavorable	28	11.161 ± 1.827	
FAB			
M1–M6	93	36.113 ± 2.885	<0.001
M7	12	8.542 ± 1.820	
Leukocyte (/μl)			
> 10,000	61	30.220 ± 2.974	0.803
≤ 10,000	44	33.631 ± 4.063	
MRD			
< 0.25 %	49	53.627 ± 3.151	<0.001
≥ 0.25 %	56	18.893 ± 2.425	
GATA4 expression			
Low <12.420	53	32.130 ± 3.385	0.769
High ≥12.420	52	34.765 ± 3.941	
GATA4 methylation			
Negative	46	39.141 ± 3.554	0.014
Positive	59	24.264 ± 3.671

**Table 4:** Cox multivariate analysis of GATA4 expression/promoter methylation and clinico-pathological features in pediatric AML

Variable	Odds ratio	EXP(B) 95 % CI	*P*
Cytogenetics			
Favo vs. Inter and Unfavo	5.894	2.412 (1.185–4.909)	0.015
MRD			
< 0.25 % vs. ≥0.25 %	16.241	5.986 (2.503–14.229)	0.000
Leukocyte (/μl)			
> 10,000 vs. ≤10,000	0.485	1.225 (0.691–2.172)	0.486
FAB classification			
M7 vs. M1–M6	6.645	2.630 (1.261–5.484)	0.010
GATA4 Expression			
Low vs. High	2.765	1.657 (0.914–3.007)	0.096
GATA4 Methylation			
Negative vs. Positive	1.885	0.661 (0.367–1.193)	0.170

In summary, our results showed firstly that the GATA4 promoter was consistently significantly methylated in leukemia cells, such as HL-60, MV4-11, SHI-1, U937, and K562 human myeloid leukemia cell lines; the expression of GATA4 was significantly lower in pediatric AML compared to NBM control samples, patients with methylated GATA4 showed lower GATA4 transcript levels compared to those without methylated; GATA4 promoter methylation was correlated with leukocyte and MRD, Kaplan-Meier survival analysis revealed a significantly shorter overall survival times in pediatric AML with GATA4 promoter methylation.

In this study, promoter methylation in Chinese pediatric AML was analyzed using NimbleGen Human DNA Methylation 385 K Promoter plus CpG Island arrays. This approach revealed significant differences in the methylation status of genes between pediatric AML and normal bone marrow samples. Previous studies have demonstrated that promoters of TFPI-2 [30] and miR-663 [31, 32] were hypermethylated in Chinese pediatric acute myeloid leukemia. Our results showed significantly greater GATA4 promoter hypermethylation in pediatric AML samples and 0/3 (0 %) in normal bone marrow samples. indicating that the GATA4 promoter is hypermethylated in AML.

GATA4 was suggested to be a tumor suppressor gene with promoter hypemethylation in various types of human cancers. The GATA4 promoter is methylated in glioblastoma [17], endometrioid carcinoma [18], ovarian cancer [19], gastric mucosa [21], colorectal carcinomas [22] and lung cancers [24]. To our knowledge, this is the first report describing the expression of GATA4 and promoter methylation status in pediatric AML. In this study, methylation-specific PCR (MSP) assays showed that the GATA4 promoter was hypermethylated in five leukemia cell lines, especially in SHI-1, HL-60, MV4-11, U937 and K562 cells). 5-Aza treatment significantly upregulated GATA4 expression in HL-60 and MV4-11 cells. Aberrant GATA4 promoter methylation was observed 15.0 % (3/20) of the NBM control samples compared to 56.2 % (59/105) of the pediatric AML samples. BGS analysis also showed that CpG islands in the GATA4 promoter were methylated in the AML samples and NBM samples were unmethylated. Analysis of GATA4 transcript levels showed that GATA4 expression was significantly decreased in AML patients compared to 20 NBM/ITP control and patients with methylated GATA4 showed lower GATA4 transcript levels compared to those without methylated GATA4. Taken together, our results show hypermethylation of the GATA4 promoter in Chinese pediatric AML for the first time.

GATA4 promoter hypermethylation is an important prognostic marker in several tumors. Kaplan-Meier analysis revealed that high methylation levels of the GATA4 promoter were significantly correlated with patient survival in oropharyngeal squamous cell carcinoma (OPSCC) [33]. In high grade serous ovarian carcinoma (HGSOC), GATA4 promoter methylation was associated with disease recurrence [34]. In this study, the prognostic significance of GATA4 promoter methylation was assessed in 105 cases of Chinese pediatric AML patients with clinical follow-up records. GATA4 promoter methylation was correlated with leukocyte counts and MRD. Kaplan-Meier survival analysis revealed significantly shorter overall survival in patients with GATA4 promoter methylation. These observations demonstrate that GATA4 promoter methylation correlates with poorer survival in Chinese pediatric AML.

The molecular function of GATA4 has been studied in certain tumors. Re-expression of GATA4 in human glioblastoma multiforme (GBM) cell lines, primary cultures, and brain tumor-initiating cells suppressed tumor growth in vitro and in vivo through direct activation of the cell cycle inhibitor P21 (CIP1). Re-expression of GATA4 also conferred sensitivity of GBM cells to temozolomide, a DNA alkylating agent currently used in GBM therapy. GATA4 reduced expression of APNG (alkylpurine-DNA-N-glycosylase), a DNA repair enzyme which is poorly characterized in GBM-mediated temozolomide resistance [23]. The potential function of GATA4 as a tumor suppressor was studied by inducing GATA4-overexpression in human colorectal cancer cell lines.GATA4 overexpression suppressed colony formation, proliferation, migration, invasion, and anchorage-independent growth of colorectal cancer cells [22].GATA4 can control expression of the anti-apoptotic factor Bcl-2 and the cell cycle regulator cyclin D2 in normal and neoplastic granulosa cells.GATA4 expression correlated with Bcl-2 and cyclin D2 expression in human and murine granulosa cell tumors (GCT). Moreover,GATA4 enhanced Bcl-2 and cyclin D2 promoter activity in murine GCT cells [35]. To date, the molecular function of GATA4 in pediatric AML is still unknown and further investigations are required to elucidate the role of GATA4 in pediatric leukemia.

## Conclusions

Epigenetic inactivation of GATA4 by promoter hypermethylation was observed in both AML cell lines and pediatric AML samples. Our study implicates GATA4 as a putative tumor suppressor gene in pediatric AML. In addition, our findings indicate that GATA4 promoter methylation correlates with leukocyte counts, MRD and significantly shorter overall survival in pediatric AML. Kaplan-Meier survival analysis revealed significantly shorter overall survival in pediatric AML with GATA4 promoter methylation but multivariate analysis shows that it is not an independent factor. However, further research focusing on the molecular mechanism underlying the role of GATA4 in pediatric leukemia is required.

## Additional file

Additional file 1: **Analysis of promoter methylation in pediatric AML using NimbleGen Human DNA Methylation 385 K Promoter Plus CpG Island Arrays.** (JPEG 777 kb)

## Abbreviations

GATA4: GATA binding protein 4
AML: Acute myeloid leukemia
MSP: Methylation specific PCR
BGS: Bisulfite genomic sequencing
NBM: Normal bone marrow
ITP: Idiopathic thrombocytopenic purpura
