# Supplementary figures and images for: Hypermethylation of the GATA binding protein 4 (GATA4) promoter in Chinese pediatric acute myeloid leukemia
Source: BMC Cancer. 2015 Oct 21;15:756. doi: 10.1186/s12885-015-1760-5 (PMC4618362; doi:10.1186/s12885-015-1760-5)

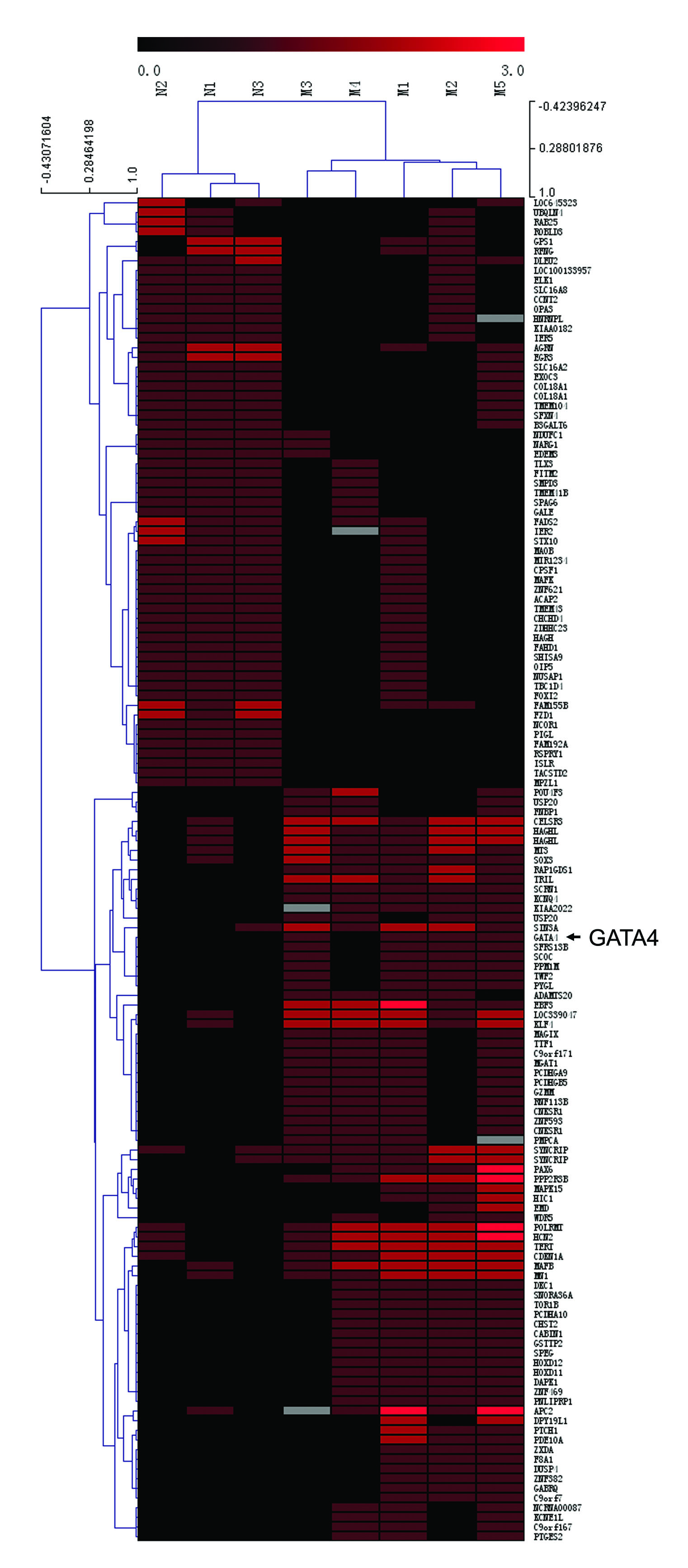

Supplement: Additional file 1: — Analysis of promoter methylation in pediatric AML using NimbleGen Human DNA Methylation 385 K Promoter Plus CpG Island Arrays. (JPEG 777 kb) [file 12885_2015_1760_MOESM1_ESM.jpeg]
